# Utility of Air Bladder-Derived Nanostructured ECM for Tissue Regeneration

**DOI:** 10.3389/fbioe.2020.553529

**Published:** 2020-10-15

**Authors:** Jianwei Wang, Jiayu Chen, Yongfeng Ran, Qianhong He, Tao Jiang, Weixu Li, Xiaohua Yu

**Affiliations:** ^1^Department of Orthopedics, The Second Affiliated Hospital, School of Medicine, Zhejiang University, Zhejiang, China; ^2^Hangzhou Huamai Medical Devices Co., Ltd., Hangzhou, China; ^3^Shanghai Key Laboratory for Prevention and Treatment of Bone and Joint Diseases, Ruijin Hospital, Shanghai Institute of Traumatology and Orthopaedics, Shanghai Jiao Tong University School of Medicine, Shanghai, China

**Keywords:** bone graft, osteogenic, ECM, composite scaffold, bone defect

## Abstract

Exploration for ideal bone regeneration materials still remains a hot research topic due to the unmet clinical challenge of large bone defect healing. Bone grafting materials have gradually evolved from single component to multiple-component composite, but their functions during bone healing still only regulate one or two biological processes. Therefore, there is an urgent need to develop novel materials with more complex composition, which convey multiple biological functions during bone regeneration. Here, we report an naturally nanostructured ECM based composite scaffold derived from fish air bladder and combined with dicalcium phosphate (DCP) microparticles to form a new type of bone grafting material. The DCP/acellular tissue matrix (DCP/ATM) scaffold demonstrated porous structure with porosity over 65% and great capability of absorbing water and other biologics. *In vitro* cell culture study showed that DCP/ATM scaffold could better support osteoblast proliferation and differentiation in comparison with DCP/ADC made from acid extracted fish collagen. Moreover, DCP/ATM also demonstrated more potent bone regenerative properties in a rat calvarial defect model, indicating incorporation of ECM based matrix in the scaffolds could better support bone formation. Taken together, this study demonstrates a new avenue toward the development of new type of bone regeneration biomaterial utilizing ECM as its key components.

## Introduction

Repair of critical-sized bone defects resulted from high-energy trauma, infection, resection of bone tumors or congenital malformation still remains a major challenge in clinic (Seal et al., 2001; El-Rashidy et al., 2017; Qu et al., 2019; Pereira et al., 2020). Although still regarded as the gold standard for bone repair treatment, autogenous bone grafts suffer from their intrinsic drawbacks such as donor site morbidity, limited availability, etc. (García-Gareta et al., 2015; Turnbull et al., 2018; Wubneh et al., 2018). Various biomaterials have been developed trying to replace autologous bone in the context of bone healing in the past three decades (Ashwin et al., 2020; Bargavi et al., 2020; Yu et al., 2020). The first generation of these materials focused on ceramics-based materials, which is represented by hydroxyapatite and other calcium phosphates (Cutright et al., 1972; Yamada et al., 1997; LeGeros, 2002; Tien et al., 2004; Ogose et al., 2005; Komaki et al., 2006; Ng et al., 2008; Fei et al., 2012; Wang et al., 2012; Liu et al., 2013). While these materials have been widely used in clinic and achieved certain successes, their applications in large bone defects are extremely challenging due to their limited bioactivity and flexibility (Woodard et al., 2007; Chocholata et al., 2019; Bohner et al., 2020). However, ideal bone tissue engineering scaffolds may require sufficient porosity with proper pore size distribution and interconnectivity, capability to facilitate cell-material interactions, as well as potency to actively regulate stem cell behaviors. Therefore, more recent researches have been exploring a composite material strategy, which employed a series of polymeric materials such as poly (lactic acid) (PLLA), hyaluronic acid, and collagen to mimic the natural composition of bone tissue (Yu et al., 2012; Ohba et al., 2016; Wang et al., 2018). In this context, the application of collagen in the composite has not only introduced a number of exciting researches, but also led to a series of commercial products including Healos by J&J, Vitoss by Stryker, etc. However, clinical applications for these products are limited to bone void filler, which can only be used for small bone defects, mainly in the non-load bearing sites. The moderate bone regenerative capability of collagen based composite largely relies on the physiochemical and biological properties of the collagen extracted from mammal tissue (Lin et al., 2019). Chemical extraction process substantially damages the natural structure including conformational structure of collagen, which led to suboptimal biological performance *in vivo*. For example, these collagen materials cannot proactively regulate growth factor interactions during bone formation (Hudalla and Murphy, 2011). Moreover, chemical crosslink of the materials is required to guarantee sufficient degradation duration, but it could inevitably introduce toxicity into the composite, leading to deteriorative effect once implanted (Yamauchi et al., 2018; Gu et al., 2019). Therefore, it is highly desirable to seek materials which can circumvent the issues associated with chemically extracted collagen.

Collagen still remains the primary choice for bone graft materials for over two decades (Horbett, 2018; Lee and Kim, 2018; Stuckensen et al., 2019), but its biological function limited collagen based bone graft materials for more challenging bone healing scenario. Collagen can only exert simple biological function such as supporting cell adhesion due to its single composition (Nakamura et al., 2019). In contrast, ECM possesses much more complex yet biocompatible composition, which allows it to regulate multiple biological processes. Extracellular matrix (ECM), a natural ideal biologic scaffold material has been widely utilized in various tissue reconstruction applications (Badylak, 2007; Chang-geng and Jian-qiang, 2011; Ma and Zhang, 2014; Jakus et al., 2017). By providing a 3D structural template and bioactive niche to cells, ECM plays various important roles during different stages of tissue regeneration (Cramer and Badylak, 2019). The key components of ECM including collagen, fibronectin, and laminin, perfectly support host cell attachment and adhesion (Costa et al., 2017). Meanwhile, component growth factors such as vascular endothelial growth factor (VEGF), basic fibroblast growth factor (bFGF) can be gradually released along with the degradation of ECM to facilitate tissue growth and remodeling (De Aza et al., 2003; Schultz and Wysocki, 2009). The degradation products of ECM might also positively impact tissue regeneration process via modulating angiogenesis and recruiting endothelial cell through certain peptides. ECM based biological materials have been successfully used to reconstruct a number of tissues such as skin, urinary bladder, ligament, and diaphragm, etc (Lin et al., 2019). Although large population of clinical successes have been achieved using ECM, its applications have been focused on soft tissues, largely due to the fact that ECM were mainly derived from soft tissues. However, as ECM possess excellent biocompatibility and potent bioactivity, its utility in other fields such as bone should definitely be explored.

Herein, we report an ECM based composite materials for large bone defect healing to demonstrate the feasibility of utility of ECM in the field of bone regeneration. We chose fish air bladder as the source of the ECM used in this study. Fish collagen, as the major component of ECM, possesses the advantages of great biocompatibility, low immunity, and controllable biodegradability (Elango et al., 2016). Moreover, the source of fish ECM is abundant and low-cost, which make it an ideal raw material for ECM based scaffold fabrication. In particular, we chose fish air bladder as our ECM source because it contains more than 80% collagen, relatively low cell population compared to other tissues such as skin, excellent mechanical strength owing to its inflation function. Air bladder ECM possess a natural nanostructure, which would play an important role during tissue regeneration (Mredha et al., 2017). We hypothesize that application of fish air bladder ECM would substantially promote bone regeneration in critical-sized bone defects due to their compositional and structural advantages. We processed air bladder tissue via a classical decellularization approach combined with micronization technique to obtain acellular tissue matrix (ATM). ATM was then combined with dicalcium phosphate (DCP) to form composite scaffold for bone regeneration. DCP microparticles were chosen for their relatively fast degradation property compared to conventional bioceramics such as hydroxyapatite and β-tricalcium phosphate (β-TCP), as well as their convenience to be incorporated into ECM network. Our results indicate that this new type of scaffolds can support osteoblast survival and differentiation, as well as promote bone healing in a rat calvarial defect model. Thus, ECM based composite scaffold might open door for the development of a new type of bone graft materials with enhanced bone regenerative properties.

## Materials and Methods

### Materials

Acellular tissue matrix was processed from silver carp fish air bladders (*Aristichthys nobilis*). All reagents were received from Solarbio and Thermo Fisher Scientific in analytical grade unless otherwise stated. Pharmaceutical grade dicalcium phosphate (CaHPO_4_) (DCP) were used as the inorganic phase in the composite at various contents in this study.

### *Aristichthys nobilis* Air Bladder Decellularization and Characterization

Silver carp (*Aristichthys nobilis*) air bladders were freshly collected from market and kept refrigerated till further use. Air bladders were firstly rinsed extensively with de-ionized water to remove residue blood and other contaminants. Air bladder tissue was blot-dried with tissue paper and weighted after cleaning. The weighted air bladder tissue (total 13 air bladders with total wet weight 140.8 g) was further treated with 600 mL of 0.2% triton X-100 solution for 3 h at room temperature with continuous agitation. The tissue was collected after extensively washing in de-ionized water and cut into 1 cm × 1 cm pieces. 200 mL of 0.9% NaCl was added into the tissue, then the mixture was transferred into a knife mill (Retsch GM 200, Germany) and grinded for 5 times at 8000 RPM for 10 s. The treated tissue was collected via centrifugation at 2500 *g* for 5 min, then re-suspended in 0.9% NaCl at 10% (w/w). The tissue suspension was further treated with 24 mM sodium deoxycholate in 0.2% EDTA overnight at room temperature with continuous agitation followed by 10 washes (5 min/wash) with 0.9% NaCl saline, then stored in freezer at −30°C for future use. The total weight of the collected decellularized bladder tissue was 118.0 g with water content about 92.30%.

The decellularization process was evaluated via a series of analytical measurements for residue DNA, fat, and collagen content quantification to guarantee the efficacy of this treatment process.

For DNA measurement, a PicoGreen assay was performed according to manufacture protocol. Briefly, both non-decellularized and decellurized bladder tissue was weighted and digested in 14.3% proteinase-K in 3% bovine albumin buffer at 60°C until completely dissolved. The treated samples were incubated with PicoGreen reagent (Thermo Fisher Scientific, United States) as guided by manufacture manual at ratio of 1 after diluted with TE buffer. The fluorescence was measured at Ex/Em of 485/530 nm using a microplate reader (Molecular Devices, United States). λ DNA provided by the manufacture was used to set up the standard curve.

Fat content was determined as previously described (Knapp and Knappová, 2013). Briefly, both untreated and treated bladder tissue were immersed in 2 M hydrochloric acid at 90°C overnight till completely dissolved. The fat was then extracted with anhydrous ether and the weight of residue fat was measured using analytical scale (Mettler Toledo, United States).

The collagen content of the bladder tissue was quantified by determining the amount of hydroproline (HYP) as HYP is an typical amino acid only specifically found in collagen with a concentration of 13.5% as reported in previous literature (Ignat’eva et al., 2007). Briefly, the treated tissue samples were incubated with 3M sulfuric acid at 105°C overnight. The resultant samples were neutralized by NaOH and oxidized with chloramine T. The samples were further reacted with *p*-dimethylaminobenzaldehyde at 60°C for 20 min. HYP concentrations were measured at 558 nm using a microplate reader (Molecular Device, United States). The collagen content was calculated based on the fixed HYP ratio of 13.5% in collagen according to previously published references (Hong et al., 2005).

Both native bladder tissue and processed bladder tissue was embedded and sectioned in paraffin sections and a hematoxylin and eosin (HE) staining was conducted to visualize the presence of any cellular components. Images of H&E staining were recorded using a Zeiss Axio Imager Z1 microscope with a RGB chromogenic filter set (Zeiss, #487933).

### DCP/ATM Composite Fabrication and Characterization

The dry weight of the decellularized air bladder tissue was determined to be approximately 90.19% (weight percent of the wet weight of bladder tissue) using a water content analyzer (XQ201, China). In order to fabricate composite scaffolds with varying dicalcium phosphate (DCP) content, DCP was mixed with decellularized tissue (dry weight) at a weight ratio of 0, 33, and 67%. Briefly, pre-calculated weight of decellularized tissue was added into the loading tank of a knife mill (Retsch GM 200, Germany), then DCP powder was added into the tank according to the predetermined ratios. After that, equal weight (total weight added in the last step) of 0.9% NaCl solution was added into each group and grinded at 6000 RPM for 10 s for 5 times with a knife mill (Retsch GM 200, Germany). The collected mixtures were then placed in a 100 mm Petri dish and further compressed into a disc shape. The samples were frozen at −30°C overnight and lyophilized for 48 h. The composite scaffolds with varying DCP contents were cross-linked with 2% 1-ethyl-3-(3-dimethylaminopropyl) carbodiimide (EDC) at 4°C for 24 h. After washing with de-ionized water, the residue cross-linker was neutralized with 0.1 M glycine solution for overnight. The scaffolds were then rinsed thoroughly with de-ionized water and lyophilized again. The dried scaffolds were cut into a size of 6 mm in diameter × 0.3 mm in length cylinder sheets using a biopsy punch.

As a head-to-head control, we also fabricated another type of composite scaffold using acid dissolved collagen (ADC) derived from the same kind of fish air bladder. The choice of this control group is to replicate the large number of collagen based bone substitute grafts widely used in both research and clinic, but originated from fish species. The collagen extraction was performed following previous study (Bama et al., 2010). After collagen reconstitution in saline buffer, the preparation of DCP/ADC composite scaffolds was following the same procedure used to fabricate DCP/ATM composite scaffolds.

The morphologies and macrostructure was examined using field emission scanning electron microscopy (DSEM, SU-3500, Japan) at 5 kV after sputter coated with gold. The scaffold porosity was calculated based on the method described by Ma and Zhang (1999). Water absorption experiments were carried out in PBS. Briefly, pre-weighed scaffolds were soak in PBS for 2 min and then taken out of the buffer and weight on balance again to determine their water absorption capability. Scaffold compositions were analyzed by the Fourier transforms infrared spectroscopy (FTIR, Nicolet, United States), the range of 4000−400 cm^–1^ with an average scan number of 128.

### MC3T3-E1 Cell Culture

MC3T3-E1 cells were cultured in alpha minimum essential medium (α-MEM) supplemented with 10% fetal bovine serum (FBS) (Corning, New York, NY, United States) and 1% pen-strep (Corning, New York, NY, United States). Cells were grown in a humidified atmosphere of 5% CO2 at 37°C. The culture medium was changed every other day. An osteogenic medium, consisting of α-MEM plus 10 mM β-glycerol phosphate and 50 mg/L-ascorbic acid (Sigma, St. Louis, MO, United States), was used for osteogenic differentiation of the cells.

### MC3T3-E1 Proliferation and Osteogenic Differentiation

MC3T3-E1 cells were seeded on to two types of scaffolds: 67%DCP/ADC and 67%DCP/ATM (Ø6 mm, 0.5 mm thickness, *n* = 4) at 200,000 cells per scaffold in 48-well plate. At day 3, 7, and 14, cell metabolic activity on each scaffold was evaluated using a CellTiter-Blue assay kit (Promega, United States) following manufacture protocol. ALP was chose as an early osteogenic marker for cell differentiation. Briefly, 200 μL cell lysis buffer with 0.5% Triton X-100 was added into each well and agitated for 20 min. The cell lysis was then collected and centrifuged at 2000 × *g* for 5 min. Aliquots of supernatants were subjected for total protein assay using a BCA assay kit (Thermo Fisher Scientific, United States) and ALP activity using an ALP assay kit (Abcam, United States). The ALP activity was expressed as total activity per microgram protein per scaffold.

After 14 days of culture, a portion of scaffolds were also collected for visualize the cell distribution within the scaffolds via actin staining and SEM examination. For actin staining, scaffolds were embedded in optimal cutting temperature (OCT) section medium and sectioned into 5 μm sections. FITC conjugated phalloidin was used as a label to show actin organization of cells in order to visualize cell distribution. Scaffolds with cells were also fixed in 2.5%glutaraldehyde buffer for 1 h. The fixed cells were then dehydrated in graded alcohol series and followed by a critical point drying. All the samples were sputter-coated with gold. Finally, the cell morphology on both types of scaffolds was examined using field emission scanning electron microscopy with acceleration voltage set at 2 kV. In order to observe the cell morphology and distribution both on the surface and interior of the scaffolds, each scaffold was cut from the middle along the sagittal direction to expose the cells residing in the interior of the scaffolds.

### Rat Calvarial Bone Defect Model

Animal experiments were approved by the Experimental Animal Welfare and Ethics Committee of Zhejiang Chinese Medical University. 36 adult male Sprague−Dawley (SD) rats (Experimental Animal Center of Zhejiang Chinese Medical University, Hangzhou, China) weighing 250∼300 g were maintained under specific pathogen-free conditions. The rats were randomly divided into three groups (*n* = 6) with two time points set at 6 and 12 week: Blank, 67% DCP/ADC, and 67%DCP/ATM. General anesthesia was induced by intraperitoneal injection with 3% pentobarbital sodium (1 mL/kg). Following anesthetization, the skull was surgically prepped and mounted onto a stereotactic restraint. After shaving and disinfection, the surgical area was incised along the midline and the subcutaneous fascia was divided until the periosteal layer was revealed. A 6 mm diameter circular defect was excavated using a dental drill. The composite scaffolds were placed into the defect loaded with 50 million rat bone marrow cells freshly isolated before surgery. The incision was then closed using biodegradable suture to allow healing.

### MicroCT Evaluation for New Bone Formation

After 6 and 12 weeks of surgery, SD rats were euthanized and the skull with the defect sites were harvested and fixed in 4% paraformaldehyde for at least 24 h. New bone healing within the defect area was evaluated using a quantitative microcomputed tomographic (microCT) (mCT40, Scanco Medical AG, Switzerland). Three-dimensional 16-bit grayscale images were reconstructed using standard convolution back-projection algorithms with Shepp–Logan filter. Based on the MicroCT results, Bone volume/tissue volume (BV/TV), bone mineral density (BMD, mg HA/cm^3^), were selected to determine the osteogenic capability of different types of composite scaffolds.

### Histology

The specimens were fixed in 10% buffered formalin for 1 week. After fixation, the specimens were decalcified in 15% EDTA (pH = 4.2) for 4 weeks, dehydrated through ethanol gradients, embedded in paraffin, and cut into 5 μm sections with a microtome for staining with hematoxylin and eosin (HE) to evaluate the regenerative properties of varying composite scaffolds. Images of H&E staining were recorded using a Zeiss Axio Imager Z1 microscope with a RGB chromogenic filter set (Zeiss, #487933).

### Statistical Analysis

The data in this study were expressed in the format of means ± standard deviation. One-way ANOVA with Tukey *post hoc* analysis was used to determine the statistical significance (*p* < 0.05) of different scaffold groups.

## Results

**FIGURE 1:**
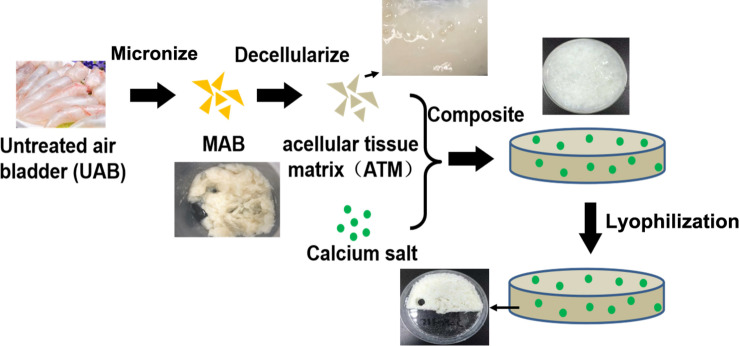
Schematic for air bladder derived composite scaffold fabrication: A Fish air bladder was cleaned then micronized using a knife mill to form microfibers. The microfibers were decellularized through a series of detergent treatment and washing steps to receive acellular tissue matrix (ATM). Next, dicalcium phosphate (DCP) microparticles were mixed and homogenized with ATM to form DCP/ATM composite. The final composite scaffold was obtained via lyophilization.

To develop a novel type of bone tissue engineering scaffold using fish derived materials, the overarching strategy was to leverage the advantages of fish air bladder tissue matrix to design an acellular tissue matrix based composite scaffold for bone regeneration. Air bladder tissue was firstly micronized into microfibers to destruct the original tissue structure; then micronized tissue was decellularized via a series of chemical treatment to remove any cellular components. Dicalcium phosphate (DCP) was chosen to serve as inorganic phase of composite scaffold to provide sufficient calcium and phosphate ions to facilitate bone regeneration (Figure 1). Via this approach, three dimensional spongy composite scaffolds were formed (Figure 1) after lyophilization. A series of physicochemical and biological assessment was conducted to fully characterize and evaluate the osteogenic potential of air bladder derived composite scaffolds.

### A Cellular Tissue Matrix (ATM) Was Obtained via Air Bladder Decellularization

**FIGURE 2:**
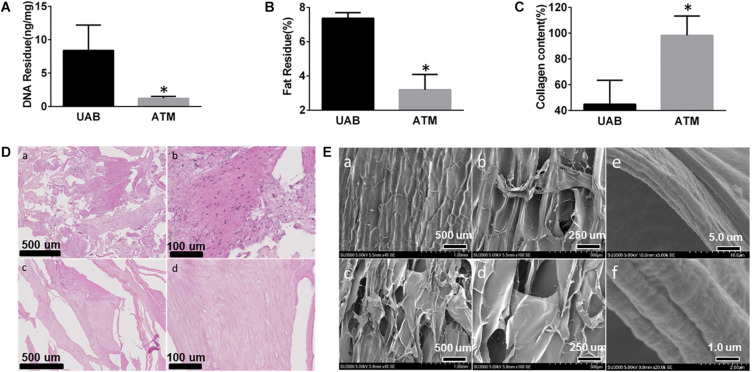
Fish air bladder decellularization efficacy evaluation: **(A)** Residue DNA before (UAB, untreated air bladder) and after (ATM, acelluar tissue matrix) decellularization, DNA residue is below 2 ng/mg, which is much lower than widely accepted standard. **(B)** Residue fat content before (UAB, untreated air bladder) and after (ATM, acelluar tissue matrix) decellularization. **(C)** Collagen content after decellularization increased from 43 to 95%, indicating clearance of most generic tissue. **(D)** HE staining of micronized air bladder tissue before and after decellularization: (a) low mag of AB tissue before decellularization. (b) High mag of AB tissue before decellularization. (c) Low mag of AB tissue after decellularization. (d) High mag of AB tissue after decellularization. **(E)** SEM micrograph of AB tissue: (a) low mag of AB tissue before decellularization; (b) High mag of AB tissue before decellularization; (c) Low mag of AB tissue after decellularization; (d) High mag of AB tissue after decellularization; (e) Low mag; and (f) High mag of collagen fibril morphology exhibiting microstructure was preserved with the collagen fibers. ^∗^Represent significant difference between UAB and ATM (*p* < 0.05).

Removal of cellular components is the key parameter for decellularized tissue matrix. Air bladder has relatively low cell density as shown in Figure 2A, the DNA content in untreated bladder tissue was only around 8 ng/mg. After decellularization, the residue DNA content in acellular tissue matrix (ATM) decreased down to 0.8 ng/mg, which is two orders of magnitude below widely accepted generic standard (50 ng/mg dry weight) (Gui et al., 2009). The treatment also demonstrated efficient removal of fat component from the tissue, which decreased from 7% down to 3% in ATM (Figure 2B). As the primary component of ECM, collagen content was determined using a hydroproline assay. After decellularization, the collagen content increased significantly from 42 to 90% (Figure 2C), indicating the decellularization process could effectively preserve the bioactive components within the matrix tissue. The maintenance of biological components was also confirmed by histological observation shown in Figure 2D. The cellular components in untreated air bladder were successfully removed after treatment (Figure 2Db,d) and no cells or cell fragments could be visualized in any regions of the processed matrix. Meanwhile, SEM micrograph of air bladder tissue before and after treatment showed no noticeable differences (Figure 2E), suggesting minimum damage to the matrix tissue during decellularization. It is noteworthy that the micrometric structure of collagen fibers in the bladder tissue was successfully preserved as shown in high magnification SEM micrographs (Figure 2Ee,f).

### ECM Composite Characterization

**FIGURE 3:**
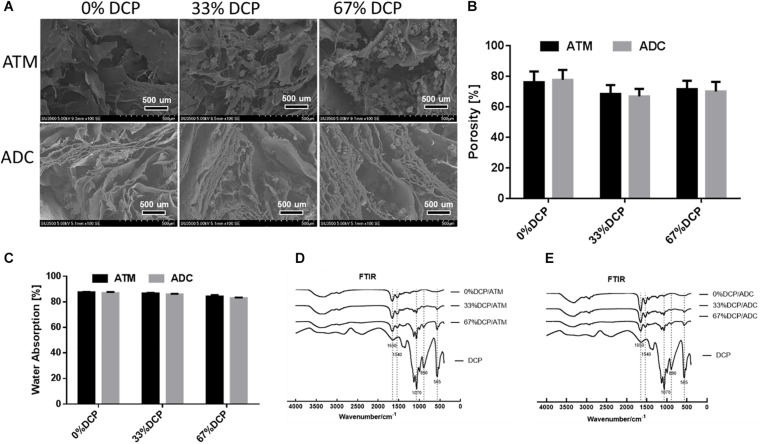
Physicochemical characterization of DCP/ATM composite scaffold: **(A)** the upper row shows SEM of ATM group with varying DCP contents; the lower row shows SEM of ADC group with varying DCP contents. **(B)** Porosity of DCP/ATM and DCP/ADC composite scaffolds with various DCP contents. **(C)** Water absorbance rate of DCP/ATM and DCP/ADC composite scaffolds with various DCP contents. **(D)** FTIR spectrum of DCP/ADC scaffolds with various DCP contents. **(E)** FTIR spectrum of DCP/ADC scaffolds with various DCP contents.

Dicalcium phosphate/ATM composite scaffolds with various DCP contents were fabricated by mixing the two components and lyophilized into 3D spongy scaffolds. As shown in Figure 3A, all scaffolds exhibited a porous network structure formed by micronized ATM. For scaffolds containing DCP, inorganic microparticles were found to be successfully incorporated among the network structure (Figure 3A). The porosity of three groups of scaffolds appeared to be around 70% and showed no significant differences between groups (Figure 3B). All types of scaffolds demonstrated capability to absorb large amount of water (Figure 3C). Both ATM and ADC incorporated scaffolds were able to absorb about 90% of the buffer after 2 min soaking in PBS, implying good body fluid absorption property once implanted *in vivo*.

The FTIR spectrums of DCP/ATM and DCP/ADC scaffolds with ratio set at 0, 33, and 67% were shown in Figures 3D,E. The absorption bands corresponding to PO_4_^3–^ group at approximately 1050–1070 cm^–1^, 870–890 cm^–1^, and 550–570 cm^–1^, which were assigned to P-O stretching mode and O-P-O bending mode, confirmed the presence of DCP in the scaffolds. When DCP content increased from 0 to 67%, the intensities of these peak gradually increased. The band around 1650 cm^–1^ arises from the C = O stretch of amide I, while the band around 1540 cm^–1^ arises from N-H deformation of amide II. The appearance of these bands in FTIR spectra of all specimen indicate that collagen serves as the major component of the composite scaffolds.

### Air Bladder Derived Composite Scaffolds Support Osteoblast Growth and Osteogenic Differentiation

**FIGURE 4:**
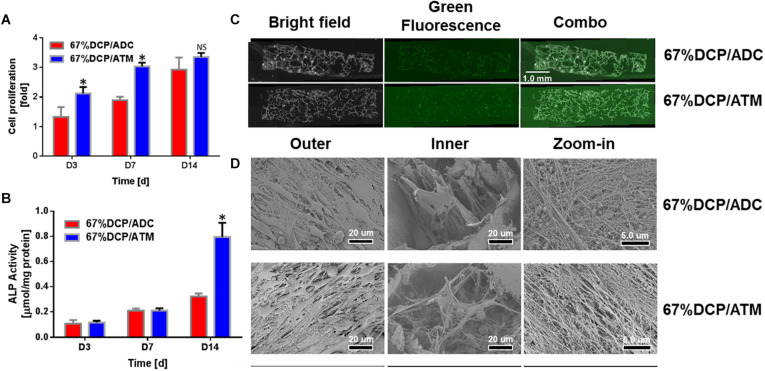
MC3T3-E1 cell *in vitro* evaluation of composite scaffolds: **(A)** The cell number increase was significantly higher on 67%DCP/ATM than on 67%DCP/ADC (*p* < 0.05) in the first week, and the cell number on both types of scaffolds showed no difference at day 14. * indicates significant differences. **(B)** ALP activity normalized to total protein content of MC3T3-E1 cultured on 67%DCP/ADC and 67%DCP/ATM for 3, 7, and 14 days. ALP activities were significantly higher on 67%DCP/ATM at day 14 (*p* < 0.05) * indicates significant differences. **(C)** Viable cell distribution on both types of composite scaffold at day 14. **(D)** SEM microgragh of MC3T3-E1 cells on 67% DCP/ADC and DCP/ATM: left column: surface; middle column: inside pores; right column: ECM deposition.

We chose to use scaffolds with DCP content set at 67% to be further evaluated both *in vitro* and *in vivo* as the weight percent of inorganic component in nature is around 65% (Hutmacher, 2000). To assess the capability of composite scaffolds to support osteoblasts proliferation and differentiation, MC3T3-E1 cells were seeded on both 67%DCP/ADC and 67%DCP/ATM and allowed to grow for 2 weeks. At day 3, the cell increasing fold on 67%DCP/ATM was 40% higher than 67%DCP/ADC. This trend continued till day 7 of cell culture. At the end of 2 weeks culture, the cell numbers on two types of scaffolds showed no significant difference, indicating the accelerated cell growth was slowed down on 67%DCP/ATM in the second week (Figure 4A). ALP was chosen as an early marker for osteogenic differentiation on the scaffolds. It was found that ALP activity on both scaffolds showed no noticeable differences during the first week of culture. However, ALP on 67%DCP/ATM jumped by more than 2-fold at day 14 of culture while its activity on 67%DCP/ADC was only increased by about 30% during the same time period (Figure 4B).

We also observed cell survival and distribution within the scaffolds. It was shown that live cells were uniformly distributed in both types of scaffold as green florescence stained for cytoskeleton actin was evenly detected throughout the scaffold thickness, however the cell density on 67%DCP/ATM was slightly higher than 67%DCP/ADC at day 14 (Figure 4C). We also visualized MC3T3-E1 cells on the scaffolds using SEM at the end of culture. It was exhibited that both types of scaffold surface were covered by large amount of cell. Cells were found residing inside the porous structure created during freeze-drying. It is noticed that the cells found in 67%DCP/ATM scaffold exhibited denser cell density on scaffold surface compared to 67%DCP/ADC. The cells residing inside the pores of 67%DCP/ATM were fully spread out with large amount of pseudopodium and deposited well organized ECM within the scaffold (Figure 4D).

### MicroCT Evaluation

**FIGURE 5:**
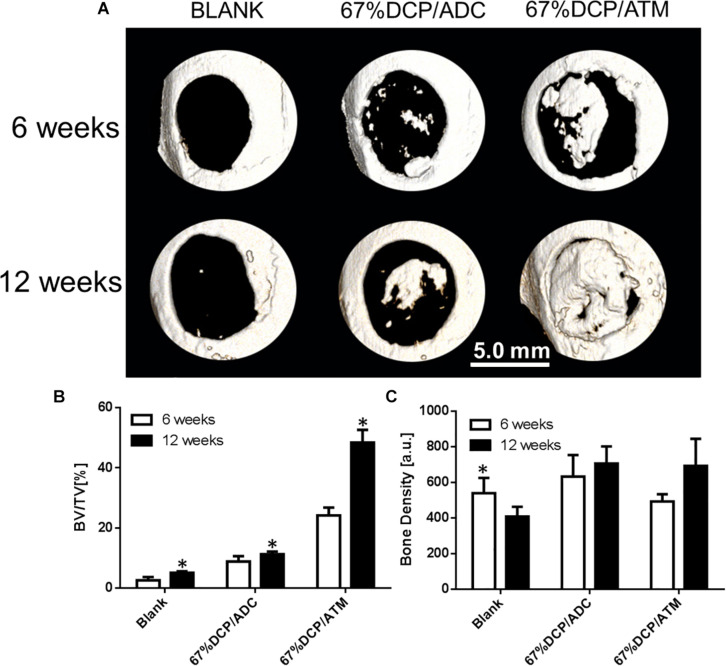
Bone healing of rat calvarial defects using three groups of treatment: **(A)** Representative micro-CT images of calvarial defects of rats 6 and 12 weeks after surgery. **(B)** BV/TV measurement shows new bone formation with 67%DCP/ATM was 50% high than 67%DCP/ADC. **(C)** Bone density shows no significant differences between two types of scaffolds. ^∗^Represent significant difference between 6 and 12 week (*p* < 0.05).

The osteogenic potential of both scaffolds was further evaluated using a rat calvarial defect model. 6 mm circular defects were created on the animal skull using a dental drill and then filled with saline (BLANK), 67%DCP/ADC, and 67%DCP/ATM, based on experimental design. The defect sites were scanned with high resolution MicroCT at 6 and 12 weeks after animals were terminated using carbon dioxide. As shown in Figure 5 at 6 weeks, the BLANK group was left empty without any defection of new bone formation at both time points. In the 67%DCP/ADC group, scattered island-like bone was formed in the defects, but it was not able to cover a portion of the defect. Meanwhile, we found a large chunk of new bone in the middle of the defect in the 67%DCP/ATM group. At 12 weeks, a noticeable area of the defect was covered by new bone tissue in the 67%DCP/ADC group. In contrast, the defect filled with 67%DCP/ATM was almost healed with new bone at this time point. We also quantified the amount of new bone by measuring BV/TV with microCT. The finds were consistent with microCT images as the BV/TV value of 67%DCP/ATM group was found to be much higher than 67%DCP/ADC group. However, when we measured the bone density in all three groups, no significant differences were found between them.

### Histology

**FIGURE 6:**
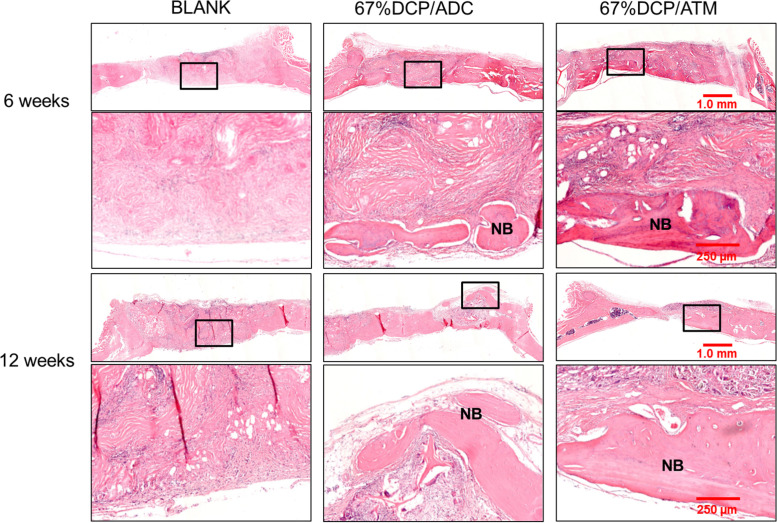
Histology of calvarial defect sites via HE staining at 6 and 12 weeks post-implantation: the 1st and 3rd row represent the whole defect view of the three tested groups: BLANK, 67%DCP/ADC, and 67% DCP/ATM; the area in the black rectangles was shown in higher magnification in the 2nd and 4th row, respectively. The 2nd and 4th row represent the high magnification view of local bone formation in each group. NB, new bone.

Histological evaluation results were correspondent with microCT imaging (Figure 6). At 6 weeks, the defect was filled with fibrous tissue without new bone shown in the blank group. In 67%DCP/ADC group, new bone formation was clearly detected at the bottom of the defect cross-section. In 67%DCP/ATM group, more new bone was observed in the defect at both the center and edge of the defect. Besides, significant more cells were detected within 67%DCP/ATM group, indicating better cell infiltration during initial stage of implantation (Figure 6, upper two rows). At 12 weeks, the BLANK group remained filled with fibrous tissue and no bone formation was found in the defect. In 67%DCP/ADC group, several bone islands were found on the edge while new bone formation also extended from the ends of the host bone. However, large area in the middle of defect was filled with fibrous tissue. The defect in 67%DCP/ATM group was almost bridged leaving a small gap in the middle of the defect. Large area of new bone grew along at the periphery of the defect, suggesting active new bone formation took place at these areas (Figure 6, lower two rows).

## Discussion

Selection of biomaterials for bone regeneration has become very diverse under the effort of biomaterials research community. Both natural and synthetic polymers have been widely used in generating various scaffolds, thin films, microsphere, and hydrogels (Solorio et al., 2015; Subramaniam et al., 2016; Murahashi et al., 2019; Wang et al., 2019). However, these materials either serve as structural support or provide cell adhesive motif during bone healing (Sarker et al., 2015; Barthes et al., 2017). Very few materials offer more complex biological functions which allow efficient regulation of multiple biological processes during tissue regeneration. In comparison, ECM as a natural cell microenvironment, can not only provide basic characteristics such as biocompatability, biodegradability, and structural template, but also possess precise nanostructure tailored for tissue ingrowth, as well as a reservoir of growth factors and cytokines (Insua-Rodríguez and Oskarsson, 2016; Theocharis et al., 2016).

Evolution of biomaterials for large bone defect healing has been continuously progressed in the past three decades. As intrinsic barriers associated with conventional approaches such as autograft could not be overcame, the focus of bone repairing materials research has gradually shifted from bioceramics (hydroxyapatite, HA), e.g., to composite materials utilizing collagen, poly (lactic acid) (PLLA), hyaluronic acid, etc. (Hutmacher, 2000; Kim et al., 2007; Niu et al., 2009; Xia et al., 2012). Even these composite materials possess relatively simple composition with two, or three major components, their roles were strictly limited to functions such as enhancing cell adhesion, promoting osteogenic differentiation, etc. during bone regeneration (Wei and Ma, 2004; Zhu et al., 2019). Thus biological materials such as ECM, which contain complex biocompatible composition may be potential candidates for novel bone repairing materials development.

Utilization of ECM for tissue regeneration requires highly efficient removal of cellular or any immunogenic components without disrupting the original structural and essential composition. Decellularization is a multi-step process including physical, chemical, and enzymatic approach (Keane et al., 2015). In this study, we reported composite scaffolds with ECM derived from fish air bladder and incorporated with dicalcium phosphate (DCP) to obtain 3D spongy scaffolds. Via a micronization technique, we successfully fabricated scaffolds with high porosity (above 65%) and fluid permeability, which allows cell ingrowth and new bone formation at the defect sites. Owing to the mild processing condition used for ATM, the natural structure of ECM was well preserved shown in Figure 2E, which might play a pivotal role once cells attach to this surface. Nano-fibrous structure possesses a high surface-to-volume ratio, which provides remarkable capability of absorbing protein and other functional molecules (Goldberg et al., 2007; Jiang et al., 2014). Nanostructure on ECM fibers was also found to proactively interact with surrounding cells and influence a series of cell behaviors such as adhere, proliferation, and differentiation (Liu et al., 2011). For instance, Lee et al. found that decellulairzed ECM nanofibers applied on PLGA strut surface promoted skeletal muscle cell orientation and maturation (Lee et al., 2019). The osteogenic capability of the ECM derived scaffolds was confirmed by healing large bone defects in a rat calvarial model. Taken together, this report open up a new avenue toward the development of novel bone repairing materials using ECM based biological tissue matrix.

Our data demonstrated that ECM based scaffolds well supported osteoblast survival and differentiation in comparison with acid derived collagen based scaffolds. When directly compared with acid dissolved collagen based scaffold, the bone formation capability provided by ATM was much more potent in our model (Figures 5, 6). ATM exhibited higher cell proliferation rate in the first week of culture as shown in Figure 4A. This results might be due to both compositional and structural differences between ATM and ADC. ATM possessed complete ECM structure. In particular, collagen in ATM was not treated by any harsh chemical condition, thus it maintained its natural conformation structure (Figure 1). Instead, ADC was extracted through an acidic process, in which collagen structure was completely broken down and lost its natural nanostructure. However, MC3T3-E1 cell proliferation rate of ADC caught up with ATM group during the second week of culture. Combined with ALP activity data (Figure 4B), it is speculated that MC3T3-E1 cells initiated osteogenic differentiation during the second week, which would greatly slow down cell proliferation, thus it was observed that cell proliferation in the two groups appeared to be similar during this time period.

When the inorganic component in the scaffold was identical, ATM could effectively induce osteogenic differentiation of osteoblast as shown in Figure 4. In comparison, Moroi et al. (2019) found that osteogenic differentiation of MC3T3-E1 cells could be modulated by adjusting the fiber diameter of self-assembled collagen fibrils from the swim bladder of Bester sturgeon. ALP activity was more effective induced using fibril with smaller nanoscale diameter (51−192 nm), which might attributed to the binding mechanism between adhesive motif on different collagen condition and cell integrins (Moroi et al., 2019). In our study, enhanced osteogenic differentiation might be owing to the rich composition of ATM. Indeed, as demonstrated in previous studies the ECM contains not only collagenous protein but also preserve significant amount of growth factors, proteoglycan, etc. (Badylak, 2007; Rowley et al., 2019). These components in ECM might play an important role during MC3T3-E1 osteogenic differentiation. In this study, we employed a chemical approach using sodium deoxycholate as main decellularization agent to remove cellular components under mild condition. Physical approach such as freeze-thawing could cause tissue matrix structure damage, which makes it inappropriate for certain applications (Arenas-Herrera et al., 2013; Rasch et al., 2019). On the other side, enzymatic approach can effectively remove DNA and RNA, its cost could be significant, which render it from clinical applications (Simsa et al., 2018). Our approach using sodium deoxycholate is approved to be a facile approach to clean cellular component in the tissue. Single cycle treatment could substantially reduce DNA content down to the required level (Figure 2). Meanwhile, our approach could also preserve the fine structure and essential components, which guarantee the quality of ECM derived from air bladder tissue (Figure 3). Besides, air bladder contains a relatively low cell density, which made it easier to achieve the acellular level required for next step use. Although compared to mammalian collagen sources, fish derived collagen possessed low immunity, low viscosity, and high fibril formation capability, its relatively low denaturing temperature leads to fast degradation both *in vitro* and *in vivo* (Moroi et al., 2019). We have circumvented this problem via decellularization instead of chemical extraction in this study; however, the properties of air bladder derived ECM requires further characterization and evaluation using both proteomes and molecular biology (Theocharis et al., 2016). For instance, we used a mouse cell line MC3T3-E1 to evaluate the scaffold *in vitro* in this pilot study, however, using primary human derived osteoblasts or mesenchymal stem cells would be more appropriate to serve our purpose, thus human primary mesenchymal stem cells will be used in our future systemic study. Besides, the role of some residue protein and other compositions have not been clearly defined in this study. Thus, while the potential of fish derived ECM has been successfully demonstrated in our study, future studies addressing the aforementioned issues need to be perform to pursue its broader applications in clinic.

## Conclusion

In this research, a novel composite scaffold DCP/ATM derived from fish air bladder and combined with dicalcium phosphate microparticles were successfully fabricated. The scaffold demonstrated porous structure with porosity over 65% and considerable capability of absorbing water and other biologics such as blood and bone marrow aspirate. *In vitro* cell culture study showed that DCP/ATM scaffold could better support osteoblast proliferation and differentiation in comparison with DCP/ADC, which is made from acid extracted fish collagen. Moreover, DCP/ATM also demonstrated more potent bone regenerative properties in a rat calvarial defect model, indicating incorporation of ECM based matrix in the scaffolds could better support bone formation. Therefore, this study demonstrate a new avenue toward the development of new type of bone regeneration biomaterial utilizing ECM as its key component.

## Data Availability Statement

The raw data supporting the conclusions of this article will be made available by the authors, without undue reservation.

## Ethics Statement

The animal study was reviewed and approved by the Experimental Animal Welfare and Ethics Committee of Zhejiang Chinese Medical University.

## Author Contributions

JW, JC, and XY performed the experiments, analyzed the data, and wrote the manuscript. YR and TJ provided the reagents and contributed to data analyses. JW, XY, and WL designed the study and contributed to data analyses. All authors read and approved the manuscript.

## Conflict of Interest

JC, YR, QH, and TJ were employed by company Hangzhou Huamai Medical Devices Co., Ltd., Hangzhou, China. The remaining authors declare that the research was conducted in the absence of any commercial or financial relationships that could be construed as a potential conflict of interest.

## Supplementary Material

Supplementary Figure 1 MicroCT images of calvarial defects at different time points.

Supplementary Figure 2 XPS examination on two types of composite scaffolds: **(A)** Typical XPS survey spectrum of 67%DCP/ADC; **(B)** Typical XPS survey spectrum of 67%DCP/ATM; **(C)** Atomic percentages (%) of different elements measured by XPS.
