# Supplementary figures and images for: Utility of Air Bladder-Derived Nanostructured ECM for Tissue Regeneration
Source: Front Bioeng Biotechnol. 2020 Oct 15;8:553529. doi: 10.3389/fbioe.2020.553529 (PMC7594528; doi:10.3389/fbioe.2020.553529)

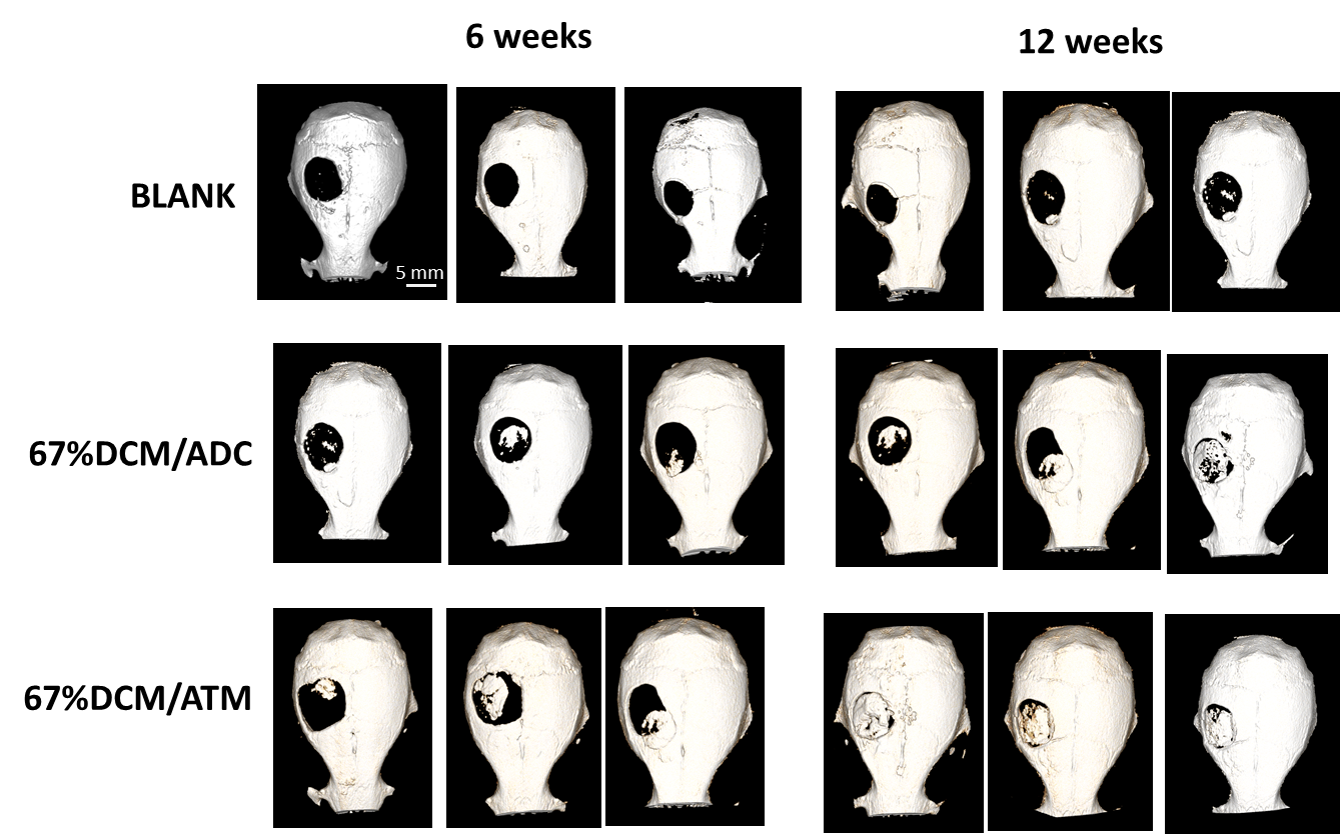

Supplement: Supplementary Figure 1 — MicroCT images of calvarial defects at different time points. [file Image_1.TIF]

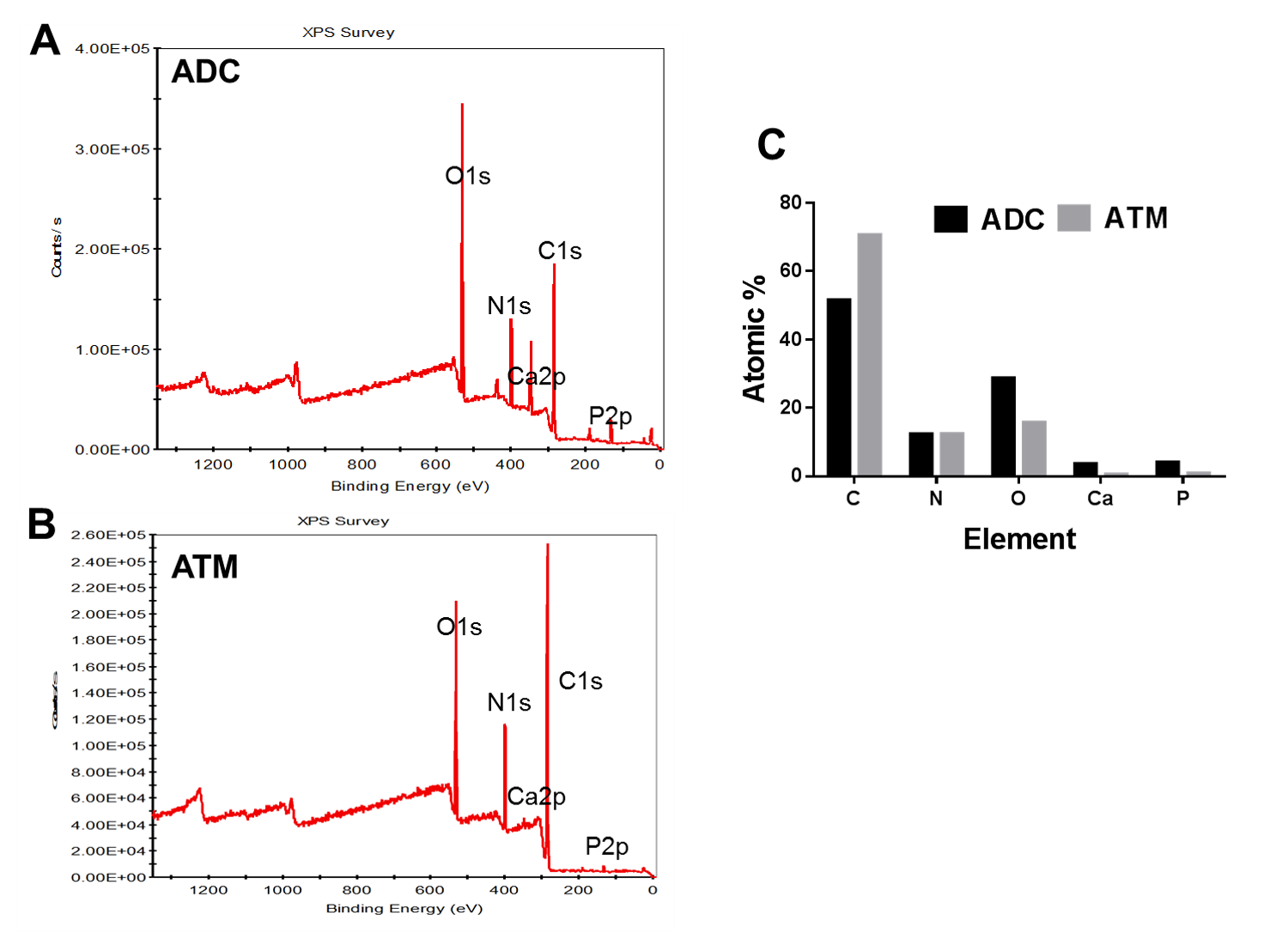

Supplement: Supplementary Figure 2 — XPS examination on two types of composite scaffolds: (A) Typical XPS survey spectrum of 67%DCP/ADC; (B) Typical XPS survey spectrum of 67%DCP/ATM; (C) Atomic percentages (%) of different elements measured by XPS. [file Image_2.TIF]
